# Supplementary material for: Simulating SARS-CoV-2 epidemics by region-specific variables and modeling contact tracing app containment
Source: NPJ Digit Med. 2021 Jan 14;4:9. doi: 10.1038/s41746-020-00374-4 (PMC7809354; doi:10.1038/s41746-020-00374-4)
Supplement: Supplementary file 1 — Supplementary file [file 41746_2020_374_MOESM1_ESM.pdf]

# Simulating SARS-CoV-2 epidemics by region-specific variables and modeling contact tracing app containment

Alberto Ferrari[1,*], Enrico Santus[2], Davide Cirillo[3,4], MiguelPonce-de-Leon[3], Nicola Marino[4,5], Maria Teresa Ferretti[4],Antonella Santuccione Chadha[4], Nikolaos Mavridis[4,6], and AlfonsoValencia[3,7]

[1] FROM Research Foundation, Papa Giovanni XXIII Hospital, Bergamo, Italy.
[2] Bayer, Decision Science & Advanced Analytics for MA, PV & RA Division.
[3] Barcelona Supercomputing Center (BSC), C/ Jordi Girona 29, 08034, Barcelona,Spain.
[4] Women's Brain Project (WBP), Gunterhausen, Switzerland.
[5] Universit`a di Foggia, Dipartimento di Scienze Madiche e Chirurgiche.
[6] Interactive Robots and Media Laboratory (IRLM), United Arab Emirates.
[7] ICREA, Pg. Llu´ıs Companys 23, 08010, Barcelona, Spain
* corresponding author: aferrari34@yahoo.com

## Supplementary Information

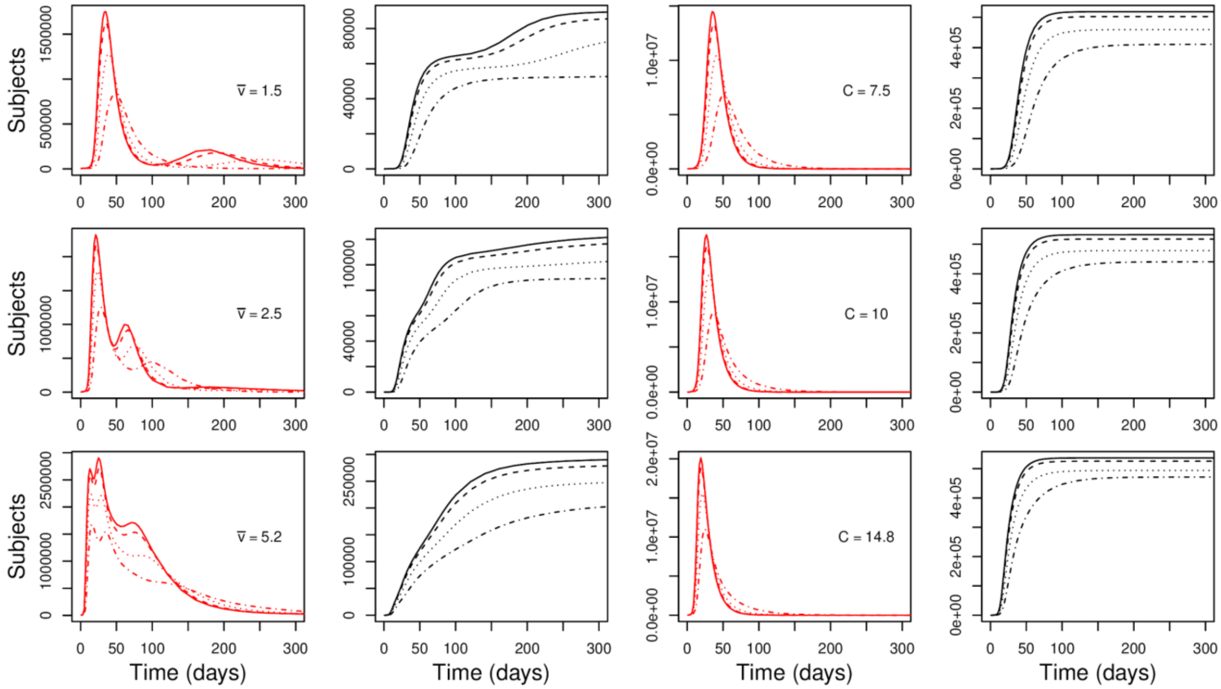


**Supplementary Figure 1**. Sensitivity analysis using f=0.5. Total symptomatic population (I+QI compartments) in red and simulated mortality (black) in the 48 scenarios. Fixed contact rate is used and reported in the right panel (plots in the first and second columns), while density-dependent contact rate is used and reported in the left panel (plots in the third and fourth columns). Each curve results from the sum over 110 districts averaged over 50 replicates per

district. Solid lines represent no app users; dashed, dotted and dashed-dotted lines show increasing fractions of the population using the app (25,50,75%).

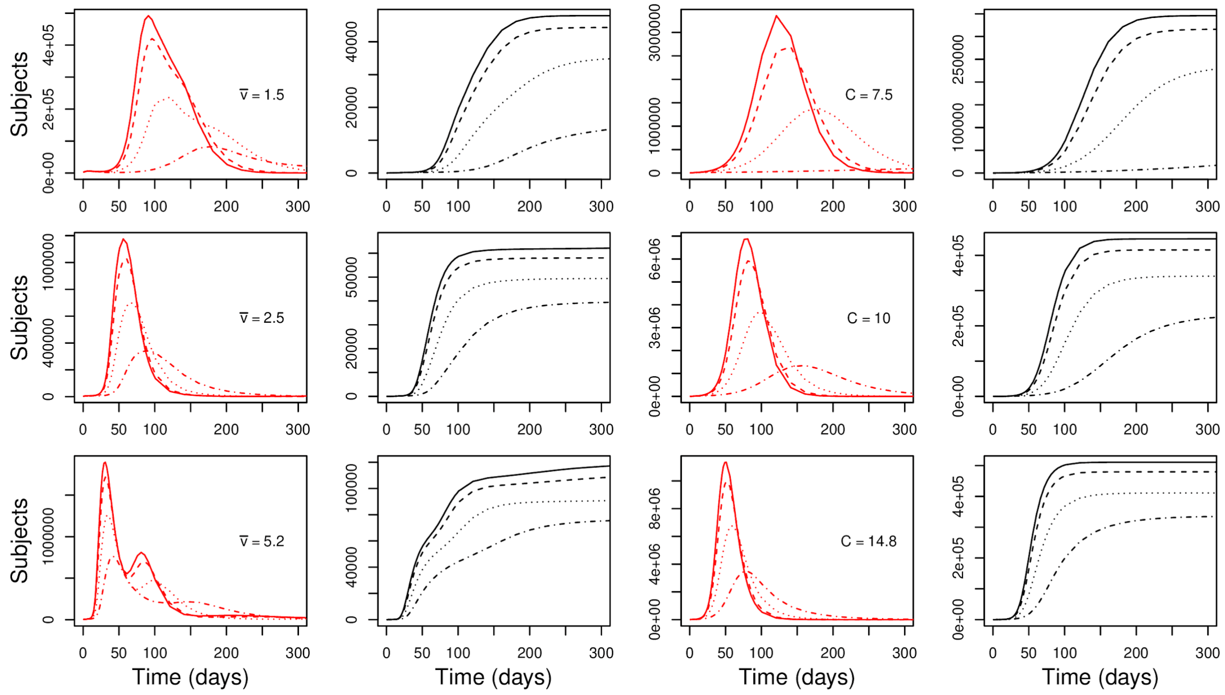


**Supplementary Figure 2**. Sensitivity analysis using $\tau_i$=7.76 (Qin J, You C, Lin Q, Hu T, Yu S, Zhou XH. *Estimation of incubation period distribution of COVID-19 using disease onset forward time: A novel cross-sectional and forward follow-up study*. *Sci Adv*. 2020;6(33):eabc1202. Published 2020 Aug 14. doi:10.1126/sciadv.abc1202). The spatial arrangement and the features of the plots (line colors and style) are the same as in Supplementary Figure 1.
